# Artificial neural network analysis of the day of the week anomaly in cryptocurrencies

**DOI:** 10.1186/s40854-023-00499-x

**Published:** 2023-05-09

**Authors:** Nuray Tosunoğlu, Hilal Abacı, Gizem Ateş, Neslihan Saygılı Akkaya

**Affiliations:** 1 Faculty of Economics and Administrative Sciences, Ankara Hacı Bayram Veli University, Ankara, Turkey; 2 Faculty of Economics and Administrative Sciences, Çankırı Karatekin University, Çankırı, Turkey; 3 Faculty of Economics and Administrative Sciences, İnönü University, Malatya, Turkey; 4 Institute of Graduate Studies, Ankara Hacı Bayram Veli University, Ankara, Turkey

**Keywords:** Cryptocurrency, Bitcoin, Ethereum, Cardano, Day-of-the-week anomaly, Artificial neural network

## Abstract

Anomalies, which are incompatible with the efficient market hypothesis and mean a deviation from normality, have attracted the attention of both financial investors and researchers. A salient research topic is the existence of anomalies in cryptocurrencies, which have a different financial structure from that of traditional financial markets. This study expands the literature by focusing on artificial neural networks to compare different currencies of the cryptocurrency market, which is hard to predict. It aims to investigate the existence of the day-of-the-week anomaly in cryptocurrencies with feedforward artificial neural networks as an alternative to traditional methods. An artificial neural network is an effective approach that can model the nonlinear and complex behavior of cryptocurrencies. On October 6, 2021, Bitcoin (BTC), Ethereum (ETH), and Cardano (ADA), which are the top three cryptocurrencies in terms of market value, were selected for this study. The data for the analysis, consisting of the daily closing prices for BTC, ETH, and ADA, were obtained from the Coinmarket.com website from January 1, 2018 to May 31, 2022. The effectiveness of the established models was tested with mean squared error, root mean squared error, mean absolute error, and Theil’s U1, and ${R}_{OOS}^{2}$ was used for out-of-sample. The Diebold–Mariano test was used to statistically reveal the difference between the out-of-sample prediction accuracies of the models. When the models created with feedforward artificial neural networks are examined, the existence of the day-of-the-week anomaly is established for BTC, but no day-of-the-week anomaly for ETH and ADA was found.

## Introduction

Cryptocurrencies started to be trading in the 2010s, depending on infrastructure and technological developments. Although they perform the same function as money, cryptocurrencies, which are created through encryption, are not dependent on national borders or central banks. In addition, although they share similar features with financial markets, fundamentally, they differ in terms of market structure (Dyhrberg et al. 2018; Maese et al. 2016). In 2012, the European Central Bank stated that cryptocurrencies do not pose a risk to financial stability due to their limited dependence on the real economy, low volume, not allowing wide user acceptance, and limited supply. However, the European Central Bank warned that cryptocurrencies should be monitored because of the surge in their volume and use in transactions. The market for cryptocurrencies has significantly evolved over the past five years since this report was released (Ceylan et al. 2018; Corbet et al. 2018a, b, c). These new digital currencies, which have gained popularity and usage day by day in the past ten years, have taken their place in markets as a new financial instrument (Dierksmeier and Seele 2018; Hudson and Urquhart 2021; Jiang et al. 2021). Cryptocurrencies have various advantages such as no taxation, low transaction and custody costs, no possibility of a seizure by any institution or organization, alternative financing source, and diversification opportunities (Kahraman et al. 2019; Omane-Adjepong and Alagidede 2019; Rejeb et al. 2021). While cryptocurrencies have emerged and developed as an innovative and efficient payment system, they also have disadvantages that can harm investors, consumers, businesses, financial systems, and even national security (Guesmi et al. 2019).

There is no consensus in the literature on the classification of cryptocurrencies, whether as money, commodities, or assets (Carrick 2016; Faria 2022; Selgin 2015; Polasik et al. 2015; Corbet et al. 2018a, b, c). The lack of consensus poses a scientific challenge in assessing the position and prospects of cryptocurrencies in financial markets (Levulytė and Šapkauskienė 2021). However, the common views of studies that categorize cryptocurrencies as money are based on the following three criteria: “It can be used for transactions, can be used as a unit of account, and can store value” (Kiyotaki and Wright 1989; Ammous 2018). First, cryptocurrencies have been increasingly used for transactions in recent years. The volume is small in comparison with other currencies. However, there are still weaker currencies with much less daily volume. The currencies of Cambodia, Laos, and Uganda are less active than Bitcoin (BTC). Furthermore, there is no specific value or volume accepted in the currency category, and this situation does not necessarily determine whether it is accepted as a medium of exchange. Second, they can be used as a unit of account. Cryptocurrencies have the unit of account features. For example, a BTC can be divided into an infinite number of parts, and these can be combined to form a complete BTC; all BTCs have the same design; and all are interchangeable. In addition, it is countable and subject to mathematical operations. Finally, the account can be used as a warehouse value. Third, users can store them for portfolio diversification purposes to accumulate value for future use, expecting that they would gain value in the future (Carrick 2016). However, studies that do not use this approach emphasize factors such as the cost of cryptocurrencies, mining, the need for intellectual capital, and anonymity (Kim 2017). In this context, the approach of governments is significant. At best, governments are indifferent to cryptocurrencies, and at worst, they oppose them because their concerns are about illegal activities. Due to the desire of governments to control economies through monetary policy, they do not want to consider cryptocurrencies in the money class as it seems to be a threat to them. For example, China has banned cryptocurrency transactions in financial institutions (Kaplanov 2012; Ponsford 2015).

This lack of a classification of cryptocurrencies is because some countries treat them as assets or commodities, whereas others consider them as a means of payment or currency (Ammous 2018). However, cryptocurrencies are no longer a niche topic in the literature but a significant trend in financial markets and an emerging asset class (Ferreira and Sandner 2021). Cryptocurrencies, which were originally supposed to act as an open-source online payment system, have evolved into a new asset class with speculative properties (Demiralay and Golitsis 2021). In the money–asset debate, cryptocurrencies support the latter view because of their high volatility, extreme short-term returns, and bubble-like price behavior (Sebastião and Godinho 2021).

This study discusses the existence of a day-of-the-week anomaly in cryptocurrencies. The day-of-the-week anomaly indicates that investors in the markets receive more returns on a certain day-of-the-week than on other days (Chatzitzisi et al. 2021). This study aims to detect the existence of the day-of-the-week anomaly in cryptocurrencies with feedforward artificial neural networks (ANNs). Unlike classical time series methods, ANN does not require prerequisites, and it gives better results than other time series method (Tosunoglu and Benli 2012; Fang et al. 2022). In this respect, it is expected that investigating the existence of the day-of-the-week anomaly in cryptocurrencies with ANN will yield more reliable results than other methods.

Most studies that have examined the day-of-the-week anomaly in cryptocurrencies were performed with classical time series analysis methods. The use of ANN in this study is a novel approach in the literature. Moreover, this study contributes to the literature by analyzing the first three cryptocurrencies—Bitcoin (BTC), Ethereum (ETH), and Cardano (ADA)—according to their market value on September 06, 2021.

## Cryptocurrencies

Cryptocurrency markets, whose use and value are increasing day by day, have grown rapidly because of the impact of innovations in money and payment systems that have been accelerated by the digitization of the economy (Brunnermeier et al. 2019; Corbet et al. 2018a, b, c; Fang et al. 2022; Niemand et al. 2021). Thus, with digitalization, new currencies have begun to shape “the nature of currency competition,” “the architecture of the international monetary system,” and “the role of public money” issued by the government (Brunnermeier et al. 2019). The most well-known cryptocurrency is BTC, which was released in 2008 under the name “BTC: Peer to Peer Electronic Cash System” and was created with blockchain technology by the person or persons named Satoshi Nakamoto (Baur et al. 2019; Kılıç and Çütcü 2018; Van Hieu et al. 2021). BTC is a point-to-point (Peer to peer (P2P)) transaction system that uses cryptography in the creation and distribution of currencies (Bhosale and Mavale 2018; Mukhopadhyay et al. 2016; Nakamoto 2008). Due to this system, payments are made directly from person to person without an intermediary.

The first BTC transaction was made by the founders of the system in January 2009. The first transaction in the real economy was made by Laszlo Hanyecz on May 22, 2010 when he bought two pizzas for 25 US dollars with 10,000 BTC (Tu and Xue 2019; Vranken 2017; Zook and Blankenship 2018).

BTC and other cryptocurrencies are a financial tool for investors to make profits due to their decentralization, anonymity, and low transaction costs, along with price volatility. However, due to the decentralized nature of the currency, consumer protection is low, and stolen BTCs can be lost forever. It also has disadvantages such as being risky due to its price being too volatile, lack of liquidity (e.g., official currencies), and being used in money laundering and smuggling transactions (Corbet et al. 2019; Fang et al. 2022; Parashar and Rasiwala 2019; Singh 2015; Sontakke and Ghaisas 2017; Viglione 2015).

BTC’s volatility and complexity initially hindered its adoption, but the advantages of blockchain—its underpinning technology—have attracted increasing attention (Xu et al. 2019). With the increase in interest in cryptocurrencies after the appearance of BTC, the crypto market has developed rapidly. There are 6,165 cryptocurrencies available today (investing.com 2021). The 10 cryptocurrencies with the highest market value are presented in Table 1. BTC has the highest market value, followed by ETH, ADA, Binance Coin, Tether, and Ripple (Table 1). The cryptocurrencies analyzed in this study are BTC, ETH, and ADA, which were the first three currencies with the highest market value as of September 06, 2021.

**Table 1:** Market capitalization and circulating offerings of the top 10 cryptocurrencies

Rank	Name	Price	Market Cap	Circulating Supply
1	Bitcoin BTC	$ 51.616,12	$ 969.672.001.184	18.808.381 BTC
2	Ethereum ETH	$3.918,58	$460.097.580.204	117.414.357 ETH
3	Cardano ADA	$2,85	$90.855.646.811	32.026.363.608 ADA
4	Binance Coin BNB	$497,26	$83.633.246.580	168.137.036 BNB
5	Tether USDT	$1,00	$67.484.059.224	67.485.377.886 USDT
6	Ripple XRP	$1,39	$63.281.482.640	46.542.338.341 XRP
7	Solana SOL	$161,83	$45.046.535.252	291.398.283 SOL
8	Dogecoin DOGE	$0,3065	$40.204.286.927	131.169.791.749 DOGE
9	Polkadot DOT	$34,23	$33.631.796.616	987.579.315 DOT
10	USD Coin USDC	$0,9998	$27.817.248.810	27.822.179.576 USDC

*Source*: (CoinmarketCap 2021) Cryptocurrency Market Capitalizations, (2021). (Accessed: September 06, 2021/ 23:26)

## Efficient market hypothesis and the day-of-the-week effect anomaly

The efficient market hypothesis put forward by Fama in 1970 is among the most researched and discussed topics in the finance literature. According to Fama, an efficient market is *“a market where there are large numbers of rational profit maximizers actively competing, with each trying to predict future market values of individual securities, and where important current information is almost freely available to all participants”* (Chuvakhin 2002).

Evaluating the market efficiency from a distributional, functional, and informational perspective, Fama stated that an efficient market completely and properly reflects the prices of financial assets with all available information in the markets at any given moment. Prices of assets are a function of information coming into the market. In an efficient market, because all investors are informed of price changes simultaneously, they cannot earn sustained and high returns as the market quickly adapts to new information. (Fama 1970; Timmermann and Granger 2004; Zeren et al. 2013).

There are three types of efficiency in the markets—weak, semi-strong, and strong forms (Eyüboğlu and Eyüboğlu 2016; Fama 1965; Sánchez-Granero et al. 2020; Santoso and Ikhsan 2020). A weak-form efficient market is when historical data and analysis created with these data do not generate income (Aksoy and Tanrıöven 2007; Lekhal and El Oubani 2020; Niroomand et al. 2020; Sewell 2012). In a semi-strong-form efficient market, investors do not have the opportunity to make excessive profits using publicly available information. Thus, in the semi-strong-form, stock prices reflect all publicly available information about a company in addition to market information, and it is not possible to make extraordinary profits with this information (Altunöz 2016; Bariviera et al. 2017; Shaker 2013; Tufan and Sarıçiçek 2013). However, in a strong-form efficient market, all public, undisclosed, and private information is reflected in the prices of financial assets. Accordingly, it is not possible to make extra profit with this information (Hawaldar et al. 2017; Ţiţan 2015).

According to the efficient market hypothesis, no investor can make an above-normal profit. However, real-life empirical findings have contradicted this hypothesis as investors can earn above-normal profits. This situation is explained by the term “anomaly,” which means deviation from the normal and is incompatible with the hypothesis.

Anomalies are briefly defined as irregularity and deviation from the general order (Frankfurter and Mcgoun 2001). Anomalies are generally examined under three headings—periodic, nonperiodic (cross-sectional), and firm and price anomalies (Erdoğan and Elmas 2010; Turaboğlu and Toplaoğlu 2017; Plastun et al. 2019). Periodic (calendar) anomalies represent the tendency of the returns of financial assets to differ on any day, week, month, or year compared with other periods (Demireli 2008; Karcıoğlu et al. 2021). Price anomalies refer to deviation from market efficiency due to overreaction and underreaction (Sümer and Aybar 2016). Among the calendar anomalies, the popular ones are the day-of-the-week, January, and year-end anomalies (Safeer and Kevin 2014). *The day-of-the-week effect anomaly* is investigated in this study.

In accordance with the day-of-the-week anomaly, while stocks or exchange rates provide higher returns on a particular day-of-the-week (usually on Fridays), lower returns are achieved on certain days (usually on Mondays) (Chiah and Zhong 2019; Rossi and Gunardi 2018; Zhang et al. 2021). One of the most common anomalies, which have many types in the literature, is the “day-of-the-week anomaly,” which particularly exists in weak-form efficient markets (Caporale and Zakirova 2017; Karan 2021; Ma and Tanizaki 2019; Plastun et al. 2019). The effect of the day-of-the-week anomaly exists because as the stock market’s last trading day is Friday, investors carry out their investments with an optimistic attitude until the last day-of-the-week and they exhibit a more hesitant attitude on the first trading day due to uncertainty. Therefore, the day-of-the-week anomaly can be determined for stocks traded on the stock market. Cryptocurrencies, which are the subject of this study, are traded 24/7 and do not have concepts such as opening and closing days. In this respect, it is important to determine whether crypto money assets, which have become the most popular investment and payment tool in recent years, provide more or less returns on any day-of-the-week. The day-of-the-week effect anomaly that can be determined in these markets can be a guide for investors.

## Literature

Due to the advantages of cryptocurrencies, their preference as an alternative investment and payment tool has been studied. Fang et al. (2022) conducted a comprehensive survey of trading cryptocurrency and a variety of other trading references. This section contextualizes our study and highlights its main contributions. The current literature on cryptocurrencies can be summarized under four main topics—research on return and volume, research on uncertainty and return, research on price, and research on the anomaly effect.

### Research on return and volume

Balcilar et al. (2017) used a nonparametric causality test to analyze the causal relationship between trading volume and BTC returns and volatility. They revealed that BTC returns and volatility based on trading volume are predictable during times other than bear and bull seasons. Nasir et al. (2019) examined the predictability of BTC volume and returns with the vector autoregression (VAR) model using Google search values ​​and determined that the frequency of Google searches leads to positive returns and an increase in BTC trading volume. Using their daily prices, Fousekis and Grigoriadis (2021) examined the relationship between trading volume and return of four cryptocurrencies—BTC, ETH, Ripple (XRP), and Litecoin (LTC)—with a cross-quantilogram. They found that low levels of trading activity generally have no informational content about future returns, while high levels tend to precede extremely positive returns. Fousekis and Tzaferi (2021) analyzed the causal link between return and volume in crypto money markets (BTC, ETH, XRP, and LTC) with the VAR model and found that returns, especially positive returns, are more likely to cause changes in traders’ expectations compared to volume.

### Research on uncertainty and return

Luo et al. (2021) examined the effect of uncertainty on BTC returns and found that investors achieved very low returns during periods of high uncertainty. Zhang et al. (2021) attempted to determine the role of downside risk in determining returns in the crypto money market through portfolio analysis and Fama–MacBeth regressions and could not find a relationship between them. Özdemir (2022) analyzed the daily closing prices of eight cryptocurrencies (BTC, ETH, Stellar, XRP, Tether, ADA, LTC, and EOS) using the E-GARCH and DCC-GARCH model and determined that volatility changed because of increasing uncertainty and risk during the coronavirus (COVID-19) pandemic.

### Research on price

Ciaian et al. (2016) examined BTC prices by considering factors specific to digital currencies using daily data and time series for five years (2009–2015). They found that market forces and BTC attractiveness for investors and users have a significant impact on the BTC price but changed over time. Poongodi et al. (2020) made price predictions of the time series of daily ether cryptocurrency closing prices using two machine learning methods—linear regression (LR) and support vector machine (SVM). The accuracy of the SVM method (96.06%) was higher than the LR method (85.46%). Using the error correction model, Hakim das Neves (2020) assessed the relationship between price and Google searches using the terms BTC, BTC crash, and crisis from December 2012 to February 2018. BTC prices are positively correlated with Google searches for BTC, which indicates the importance of attractiveness variables in determining BTC's value.

### Research on the anomaly effect

Caporale and Plastun (2019) examined the day-of-the-week effect in the cryptocurrency market with many statistical techniques (Student t-tests, regression analysis with dummy variables, Kruskal–Wallis test, ANOVA, and mean analysis) and a trade simulation approach. Although no anomalies were observed in LTC, XRP, and Dash returns, they found that BTC returns peaked on Mondays. Using the ordinary least squares (OLS) and restricted least squares models, Kurihara and Fukushima (2017) examined the effect of the day-of-the-week on BTC prices from 2010 to 2016 and found weekend anomalies (highest returns) in BTC prices. Eyüboğlu and Eyüboğlu (2016) analyzed the effects of the day-of-the-week and the month of the year in the BTC and LTC markets from 2013 to 2016 using a GARCH (1,1) model. They found that Monday, Tuesday, and Friday have a positive effect on BTC returns, while Saturday has a negative effect on LTC returns. In addition, they found that February, October, and November have positive effects on BTC returns, while August has a negative effect on LTC returns. Durai and Paul (2018) examined the effect of the day-of-the-week on BTC returns using the Ljung Box Q test on the time series of 2010–2018 data and found that the effects of Mondays and Tuesdays on BTC returns are higher, and the effects of Wednesday and Thursday on BTC returns are less. Dorfleitner and Lung (2018) analyzed eight cryptocurrencies using the EGARCH model and found that returns were lower on Sunday.

Using GARCH and OLS models, Aharon and Qadan (2019) analyzed data from 2010 to 2017 to investigate the effect of the day-of-the-week on the BTC market and stated that BTC had the highest return on Mondays. Baur et al. (2019) investigated the effects of time of day, day-of-the-week, and month of the year on BTC returns and transaction volume, finding that transaction volume decreased at the end of the week and that the anomaly effect was not permanent. However, using the student’s t-test, Decourt et al. (2019) observed that BTC returns reached the highest level on Tuesdays and Wednesdays. Yaya and Ogbanna (2019) examined 2015–2019 data with dummy variables and concluded that the effect of the day-of-the-week on BTC returns is insignificant. Robiyanto et al. (2019) examined the effect of the day-of-the-week and the month of the year on BTC and LTC from 2014 to 2018 using a GARCH model. They concluded that investors should buy BTC at the end of January and sell it at the end of February and that Monday, Wednesday, and Thursday have a positive impact on BTC returns. Kaiser (2019) tried to determine the market efficiency for ten cryptocurrencies by analyzing the seasonal effects and revealed the existence of the weak-form efficient market hypothesis.

Regarding studies carried out in the last two years, Evci (2020) analyzed 2013–2019 data with the asymmetric GARCH model and found that Monday, Thursday, and Sunday have a negative effect on BTC returns. The most losses occurred on Thursday. On the other hand, Yılmaz and Akkaya (2020) analyzed data from 2013 to 2020 with the Kruskal–Wallis-H test and could not detect the effect of the day-of-the-week on BTC returns. Nur and Dewangkara (2021) examined the day-of-the-week effects on returns and volatility on BTC, ETH, XRP, LTC, and Tether currencies using five autoregressive conditional heteroskedasticity (ARCH) models (ARCH, generalized ARCH (GARCH), exponential generalized ARCH (EGARCH), threshold ARCH (TARCH), and power ARCH (PARCH)). They found that the best model for BTC is Power ARCH (PARCH) and that Monday, Tuesday, and Friday have a positive effect on BTC returns. The best model for LTC is PARCH, and Monday and Wednesday have a negative impact on LTC returns. The best model for the ETH market is PARCH, and Tuesday has a positive effect on ETH returns, whereas Wednesday and Thursday have a negative effect on it. The best model for Tether is PARCH, and Tuesday and Friday have a positive effect on Tether yields, whereas Wednesday has a negative effect. For XRP, they revealed that the best model is TARCH, and XRP returns are also affected by Wednesday and Thursday.

Orhan et al. (2021) investigated the effect of the day-of-the-week on BTC returns using the 2,877th day closing price with ARCH and EGARCH models and observed that the day with the highest return was Monday. According to Ghaiti (2021), Wednesday has a negative effect on BTC volatility. He found that Saturdays and Sundays have a positive effect on BTC Cash and that Saturdays have a positive effect on ETH. Kinateder and Papavassiliou (2021) used a GARCH dummy model to examine the impact of calendar effects on BTC’s daily return and volatility from 2013 to 2019. They found that there is no classic DOW effect on BTC returns, but BTC’s volatility was higher at the beginning of the week. They also found a very low risk during the weekend, and the risk of investors decreased significantly in September. Qadan et al. (2022) analyzed the reaction of the daily price data of eight cryptocurrencies (BTC, ETH, LTC, XRP, Dash, Monero, Nem, and ETH Classic) to different seasonal anomalies (Saint Patrick’s Day, Valentine’s Day, Yom Kippur, Christmas, Diwali, etc.), finding that the majority of the effects examined were not present for these cryptocurrencies. They revealed that the Monday effect on BTC primarily appeared in the first three weeks.

## Methodology

The objective of this study is to investigate the day-of-the-week anomaly in the prediction of cryptocurrencies with ANN. The day-of-the-week anomaly refers to a phenomenon that implies that investors tend to earn more returns on a particular day-of-the-week than on other days (Chatzitzisi et al. 2021). This study examines whether the predictions of crypto money values made using the values of the past period differ from those made by including the day-of-the-week dummy variables.

ANN can learn both linear and nonlinear structures of time series. Therefore, analysis can be conducted without investigating the structure of the time series. An ANN is known to provide better results than traditional methods (Günay et al. 2007; Tosunoglu 2021). Thus, in this study, the day-of-the-week anomaly in cryptocurrencies is investigated with feedforward ANN. For this, a prediction model based on the lagged time series of the daily values of cryptocurrencies is compared with a second prediction model based on the delayed time series by adding the dummy variables of the day-of-the-week.

**Fig. 1:**
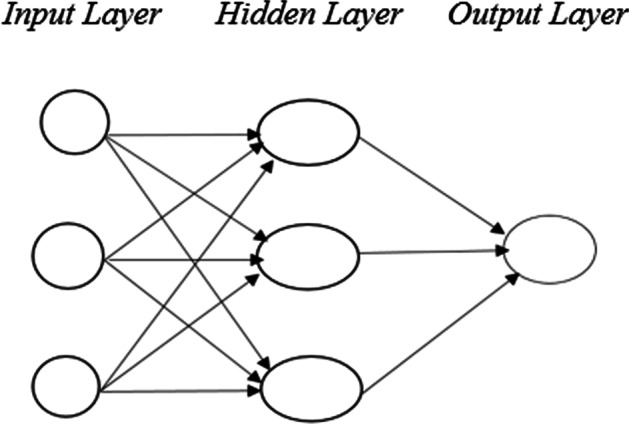
ANN structure

An ANN is a system of interconnected neurons. It consists of three layers (Fig. 1). The first layer (the input layer) contains input variables, and the last layer (the output layer) contains output variables. The hidden layer—located between these two layers—processes the information from the input layer and transmits it to the output layer. The number of these layers and neurons in the layer form the architecture of the network (Fausset 1994; Güneri and Apaydın 2004).

Each neuron in the input layer is connected to each neuron in the hidden layer with randomly determined weight coefficients. The neurons in the hidden layer are likewise connected with neurons in the output layer. Information is transmitted through these links. The transmission direction of information is divided into two—forward and backward. In feedforward networks, information is transmitted forward, while in feedback networks, information can be processed in forward, backward, and self-transmission formats (Elmas 2003; Öztemel 2003). The net input information coming to a neuron partaking in any layer is calculated through an aggregation function. Each neuron produces the output value by applying an activation function to this net value. The output value of the neuron in the output layer will be the output value of the network. The difference between the actual output value and the output value obtained from the network is the error. The performance of the network is evaluated by calculating the error function with the error value obtained from each neuron. In this study, the mean squared error (MSE) criterion is used to measure performance. The mesh weights should be changed if the error value exceeds a certain threshold. This process is termed network training. There are different learning algorithms that calculate this change in weight. A backpropagation algorithm based on the Levenberg–Marquardt algorithm is used in this study. The purpose of the Levenberg–Marquardt algorithm is to minimize the following error function:

1 $$E = \frac{1}{2}\mathop \sum \limits_{p = 1}^{n} \left( {y_{p} - \hat{y}_{p} } \right)^{2} = \frac{1}{2}\mathop \sum \limits_{p = 1}^{n} \left( {e_{p} } \right)^{2}$$

${e}_{p}$ represents the error of each observation value. The derivatives of $E$ can be written using the Jacobian matrix. The Jacobian matrix is as follows:

2 $$J\left( x \right) = \frac{{\partial e_{p} }}{{\partial W_{j} }}$$

The Levenberg–Marquardt algorithm calculates $\Delta W\left(k\right)$ with Eq. (3) to find the optimum weights that will minimize the $E$ function.

3 $$\Delta W\left( k \right) = - \left[ {J_{k}^{T} J_{k} + \lambda_{k} I} \right]^{ - 1} J_{k}^{T} e_{k}$$

In Eq. (3), $\Delta W\left(k\right)$ is the change in mesh weights over the period; *J* denotes the denotes Jacobian matrix λ the Marquardt parameter; $I$ denotes the unit matrix; and ${e}_{k}$ is the error value in the period. The Jacobian matrix defined by Eq. (2) is the matrix consisting of the first derivatives of the error function. λ is a parameter that can be changed during the algorithm. If the sum of squares error decreases in one period, λ is multiplied by a certain decay rate for the next period. Otherwise, λ is used by dividing it by the selected decay rate value for the next period. Thus, it aims to improve the performance of the network at each step.

As a result, the change in weight is given as follows (Okkan et al. 2018; Ranganathan 2004):

4 $$W\left( {k + 1} \right) = W\left( k \right) + \Delta W\left( k \right)$$

The value of the error function when it is less than the specified threshold value terminates network training. The estimates are obtained by using the best weight values.

A feedforward three-layer model will be used in the analysis of time series with ANN. The number of inputs in the input layer is the lagged values of the time series. The output layer contains the values of the time series. In this study, it is analyzed using two different ANN models for each currency. Mallqui and Fernandes (2019) suggested that a seven-day lag time should be employed in the analysis as seven cryptocurrencies are traded every hour and every day. Therefore, the number of delays is seven. The proposed models are arranged as follows:

Model 1_M(1*):*$$y\left(t\right)=f(y\left(t-1\right), ., y\left(t-7\right))$$

Model 2*_*M(2)*:* $y\left(t\right)=f(y\left(t-1\right), ., y\left(t-7\right), {D}_{1}, {D}_{2}, {D}_{3}, {D}_{4},{D}_{5},{D}_{6})$where $y\left(t\right)$ is the output value for cryptocurrencies in period t; $y\left(t-i\right)(i=1,\dots ,7)$ denotes the delayed series of cryptocurrencies; ${D}_{1}, { D}_{2}, { D}_{3}, { D}_{4}, {D}_{5}, and{ D}_{6}$ are dummy variables for Monday, Tuesday, Wednesday, Thursday, Friday, and Saturday, respectively (Sunday is the base).

In the first model, the estimation model is established based on only the lagged time series, while in the second model, the analysis is performed by adding the day-of-the-week dummy variables to the lagged series. The results of the two models will reveal whether the day-of-the-week is effective in the forecast. Here, performance criteria and R^2^ value are compared.

**Fig. 2:**
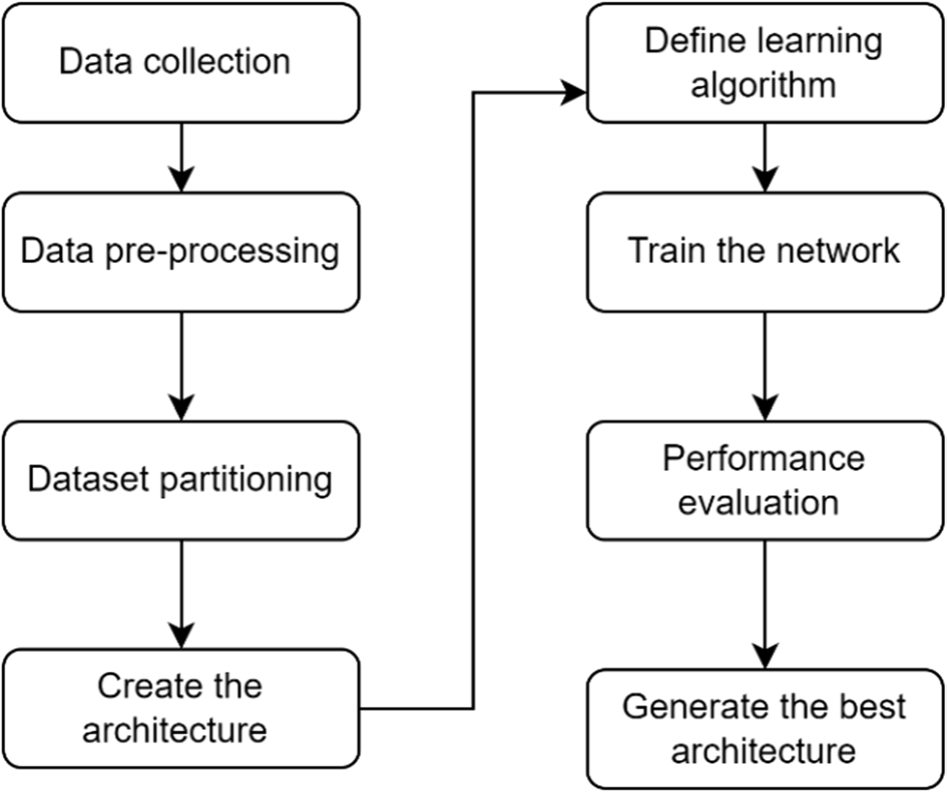
Overview of the ANN methodology

The ANN methodology of the study can be summarized as follows: (1) data collection, (2) data preprocessing, (3) data partitioning, (4) the creation of the architectural structure, (5) the selection of the learning algorithm, (6) training of the network, (7) evaluation of performance, and (8) creation of the best model. The graphical representation of the methodology is depicted in Fig. 2. Matlab R2021a_Neural Network Fitting toolbox is used for ANN analysis in the study.

### Data collection

**Fig. 3:**
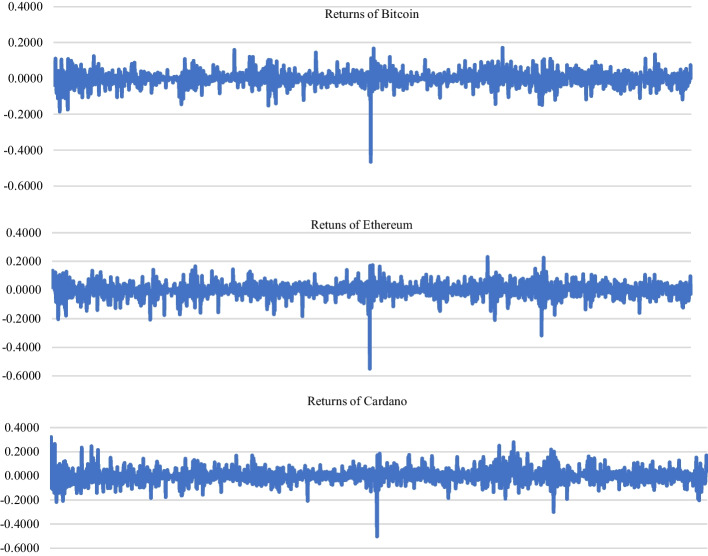
Daily returns over the sample period

In this study, daily closing prices of BTC, ETH, and ADA cryptocurrencies, which are the three highest cryptocurrencies by market value, are used as a dataset. The dataset for BTC, ETH, and ADA is from Monday, January 1, 2018 to Wednesday, May 31, 2022. The data are obtained from the “coinmarketcap.com” website. The total number of items (number of data) in the dataset is 1,611. In line with the objective of this study, return series were calculated. In this study, the daily return of each currency ${R}_{t}$ is defined as follows:

5 $$R_{t} = ln\left( {\frac{{P_{t} }}{{P_{t - 1} }}} \right)$$

where ${P}_{t}$ refers to the closing price at time t; ${R}_{t}$ refers to the return on time t; and $ln{P}_{t}$ and $ln{P}_{t-1}$ represent the natural logarithmic price at t and t − 1, respectively. The descriptive statistics of the returns are presented in Table 2, and the time series graph of daily returns over the sample period is depicted in Fig. 3.

**Table 2:** Descriptive statistics of BTC, ETH, and ADA daily return

Daily return								
	All-Day	Mon	Tue	Wed	Thurs	Fri	Sat	Sun
*Bitcoin*								
N	1611	231	230	230	230	230	230	230
Min	− 0.4647	− 0.1740	0.1846	− 0.1481	− 0.4647	− 0.1096	− 0.1135	− 0.10615
Max	0.1718	0.1718	0.1600	0.1198	0.1671	0.1448	0.1218	0.11993
Mean	0.0005	0.0019	− 0.0008	0.0028	− 0.0049	0.0031	0.0030	− 0.0016
Std. Deviation	0.0397	0.0441	0.0399	0.0409	0.0518	0.0370	0.0272	0.0325
Skewness	− 1.070	0.062	− 0.494	− 0.215	− 2.992	0.290	0.168	− 0.521
Kurtosis	13.486	2.319	3.549	1.745	27.062	1.867	4.084	2.632
*Ethereum*								
N	1611	231	230	230	230	230	230	230
Min	− 0.5507	− 0.1789	− 0.2038	− 0.3174	− 0.5507	− 0.1598	− 0.1467	− 0.1699
Max	0.2307	0.2256	0.1449	0.1294	0.1734	0.1702	0.1153	0.2307
Mean	0.0005	− 0.0006	− 0.0008	0.0015	− 0.0065	0.0031	0.0065	0.0009
Std. Deviation	0.0512	0.0540	0.0504	0.0548	0.0638	0.0481	0.0369	0.0462
Skewness	− 1.063	0.002	− 0.328	− 1.172	− 2.761	0.088	− 0.155	− 0.081
Kurtosis	10.636	2.219	2.132	5.138	23.175	1.579	2.241	4.443
*Cardano*								
N	1611	231	230	230	230	230	230	230
Min	− 0.5037	− 0.1925	− 0.2174	− 0.3011	− 0.5037	− 0.1551	− 0.1447	− 0.1457
Max	0.3221	0.2351	0.1856	0.3221	0.2455	0.2652	0.2156	0.1697
Mean	− 0.0001	− 0.0035	− 0.0014	0.0035	− 0.0105	0.0032	0.0092	− 0.0011
Std. Deviation	0.0608	0.0601	0.0592	0.0714	0.0719	0.0578	0.0508	0.0489
Skewness	− 0.059	0.170	− 0.215	0.263	− 1.042	0.815	0.894	− 0.065
Kurtosis	5.237	1.434	1.627	3.784	10.375	2.436	3.121	1.051

According to the results presented in Table 2, the lowest return of the whole week for BTC, ETH, and ADA is on Thursday. High returns are on Monday for BTC, Sunday for ETH, and Wednesday for ADA.

Figure 3 depicts the changes in logarithmic returns. The most striking change is the large negative change observed in all three currencies on the same date (March 12, 2020). The World Health Organization declared COVID-19 as a global pandemic on March 11, 2020 (Zhang et al. 2020). After this announcement, although the exact global economic effects are not yet clear, like other financial markets, the cryptocurrency markets reacted with dramatic movements (Mazur et al. 2021; James et al. 2021). A constantly changing financial environment, one of the key features of financial data, can easily affect it (Li et al. 2021).

Out-of-sample is used for performance evaluation in this study. The dataset is divided into two sets—in-sample and out-of-sample. In-sample data are used to establish the estimation model, and out-of-sample data are used to measure the effectiveness of the established model.

In the out-of-sample selection, the heuristic approach is used by examining the time graph of the data. As depicted in Fig. 3, there was a big break on March 12, 2020. Based on this date, the sample was divided into two as follows:

In-Sample Period: January 1, 2018 to March 11, 2020 (number of data: 801).

Out-of-Sample Period: March 12, 2020 to May 31, 2022 (number of data: 811).

### Data preprocessing

The data are preprocessed by using their returns for analysis.

### Dataset partitioning

In this study, we partitioned 70% of the dataset as training, 15% as validity, and 15% as the test set, using 561 for training, 120 for validity, and 120 for testing.

### Creating the architecture

All models created in the study have a feedforward three-layer network structure consisting of a single input layer, a single hidden layer, and a single output layer. There is a neuron in the output layer of the models. For each cryptocurrency, the number of neurons that will be included in the input layer is 7 for Model 1 and 13 for Model 2. Different architectures have been tested to determine the number of neurons in the hidden layer. Following the suggestion of Laboissiere et al. (2015), six network models in which the number of neurons is 5, 10, 15, 20, 25, and 30 were evaluated for each model.

### Define the learning algorithm

The learning algorithm is chosen as the backpropagation algorithm based on the Levenberg–Marquardt backpropagation algorithm. The activation function used to train the network is the sigmoid function; the output function is the linear function; the learning rate is 0.001; and the performance criterion is the mean absolute error (MAE).

### Train the network

At this stage, an output from each model is obtained by running the network. When the network is first to run, it obtains an output by making forward calculations. The error is measured using these output values and the targeted output values. If the error value is less than the initial critical value determined at the beginning, the network stops the calculations. If the error is bigger, the mesh weights are modified based on the backpropagation algorithm. The network reproduces the output using the new network coefficients obtained. This process continues until the network reaches a small error value that is less than the critical value.

### Performance evaluation of the network

At the end of the network training, the best model is selected by comparing the MSE values of the test set, the model with hidden neurons, and the model with a minimum error. The obtained results are presented in Table 3.

**Table 3:** MSE for test set

Hidden Neuron	Bitcoin M(1)	Bitcoin M(2)	Ethereum M(1)	Ethereum M(2)	Cardano M(1)	Cardano M(2)
5	2.20e−3	1.49e−3	3.83e−3	2.41e−3	3.04e−3	3.77e−3
10	1.66e−3	1.23e−3	2.68e−3	2.44e−3	3.79e−3	3.58e−3
15	1.77e−3	1.78e−3	**2.15e**−**3**	2.34e−3	4.88e−3	4.59e−3
20	1.54e−3	1.88e−3	3.19e−3	**2.17e**−**3**	**2.92e**−**3**	4.05e−3
25	**0.93e**−**3**	**1.23e**−**3**	2.89e−3	4.55e−3	4.01e−3	**3.26e**−**3**
30	1.43e−3	2.08e−3	2.56e−3	3.63e−3	4.69e−3	4.98e−3
Architecture	**7–25-1**	**13–25-1**	**7–15-1**	**13–20-1**	**7–20-1**	**13–25-1**

Bold indicates the minimum error

### Generate the best architecture and results

The results obtained at the end of the training according to the best models created are summarized below for each cryptocurrency and model.

#### Results for BTC

**Fig. 4:**
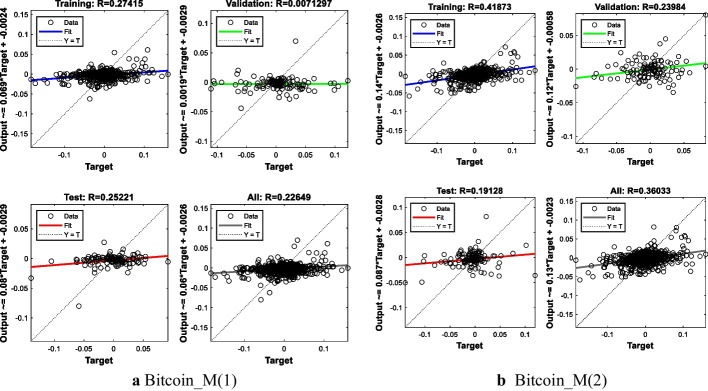
ANN training regression for BTC

The MSE value of the first model is 0.0009, and the MSE value of the second model is 0.0011. The R^2^-value is close to 1% for both models. These results indicate that the two models give similar predictions, i.e., one is not superior to the other. The results are presented in Table 4 and Fig. 4.

**Table 4:** BTC Modeling results

	Model	Network architecture	MSE for all model	R^2^ for all model	R^2^ for test set
Bitcoin	M(1)	7-25-1	0.0009	0.0513	0.0636
	M(2)	13-25-1	0.0011	0.1298	0.0365

#### Results for ETH

**Fig. 5:**
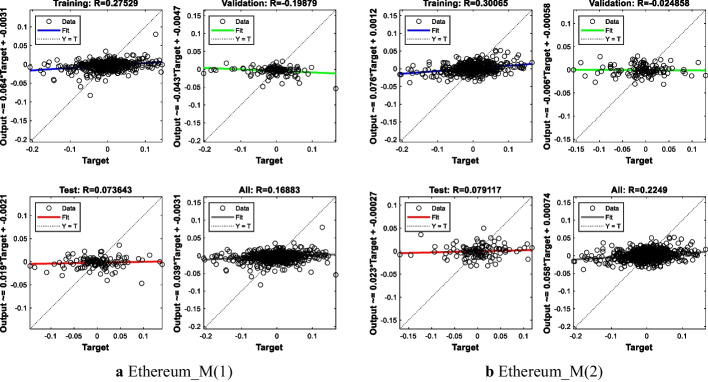
ANN training regression for ETH

The MSE value of the first model is 0.0018, while the MSE value of the second model is 0.0018. The R^2^-value is close to 1% for both models. These results indicate that the two models give similar predictions and that one is not superior to the other. The results are presented in Table 5 and Fig. 5.

**Table 5:** ETH modeling results

	Model	Network architecture	MSE for all model	R^2^ for all model	R^2^ for test set
Ethereum	M(1)	7-15-1	0.0018	0.0170	0.0054
	M(2)	13-20-1	0.0018	0.0506	0.0062

#### Results of ADA

**Fig. 6:**
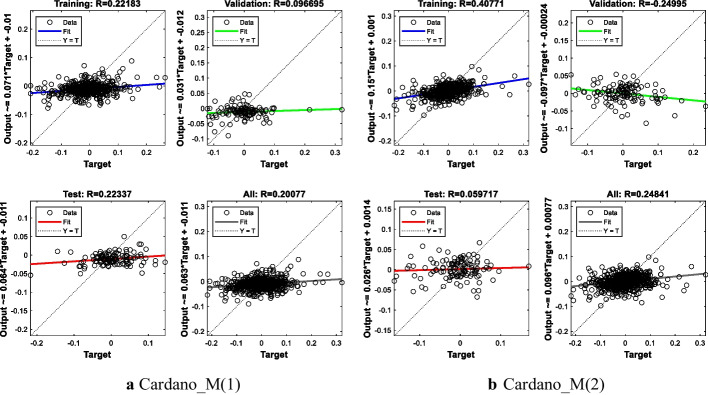
ANN training regression for ADA

The MSE value of the first model is 0.0026, while the MSE value of the second model is 0.0022. The R^2^-value is close to 1% for both models. These results indicate that the two models give similar predictions and that one is not superior to the other. The results are presented in Table 6 and Fig. 6.

**Table 6:** ADA modeling results

	Model	Network architecture	MSE for all model	R^2^ for all model	R^2^ for test set
Cardano	M(1)	7-20-1	0.0026	0.0403	0.0499
	M(2)	13-25-1	0.0022	0.0617	0.0036

### Evaluation of out-of-sample

In addition to the error and verification indicators of each model, the success of the network is tested using out-of-sample data selected from the dataset at the beginning of the study and not previously known by the network. The descriptive statistics of the out-of-sample are presented in Table 7.

**Table 7:** Descriptive statistics of the out-of-sample set

		Minimum	Maximum	Mean	St. Deviation
Bitcoin (n = 810)	Target	− 0.4647	0.1718	0.0017	0.0410
	M(1)	− 0.0401	0.0108	− 0.0028	0.0033
	M(2)	− 0.0432	0.0122	− 0.0027	0.0036
Ethereum (n = 810)	Target	− 0.5507	0.2307	0.0028	0.0531
	M(1)	− 0.0126	0.0023	− 0.0020	0.0010
	M(2)	− 0.0129	0.0050	− 0.0002	0.0012
Cardano (n = 810)	Target	− 0.5037	0.2794	0.0034	0.0632
	M(1)	− 0.0432	0.0068	− 0.0108	0.0012
	M(2)	− 0.0117	0.0087	− 0.0015	0.0016

Performance criteria were calculated by using the out-of-sample set for the performance evaluation of the models. The performance criteria used in this study are MSE, root mean squared error (RMSE), MAE, and Theil’s U_1_, and ${R}_{OOS}^{2}$ is used for out-of-sample test (Gu et al 2020). Performance criteria ${r}_{t}$, out-of-sample return value at time t and ${\widehat{r}}_{t}$, and forecast return at time t were calculated using the following equations, and the results are presented in Table 8.

6 $$MSE = \frac{{\mathop \sum \nolimits_{t = 1}^{N} (r_{t} - \hat{r}_{t} )^{2} }}{N}$$

7 $$RMSE = \sqrt {\frac{{\mathop \sum \nolimits_{t = 1}^{N} (r_{t} - \hat{r}_{t} )^{2} }}{N}}$$

8 $$MAE = \frac{{\mathop \sum \nolimits_{t = 1}^{N} \left| {r_{t} - \hat{r}_{t} } \right|}}{N}$$

9 $$Theil^{^{\prime}} s U_{1} = \frac{{\sqrt {\frac{1}{N}\mathop \sum \nolimits_{t = 1}^{N} \left( {r_{t} - \hat{r}_{t} } \right) ^{2} } }}{{\sqrt {\frac{1}{N}\mathop \sum \nolimits_{t = 1}^{N} \left( {r_{t} } \right) ^{2} } + \sqrt {\frac{1}{n}\mathop \sum \nolimits_{t = 1}^{N} \left( {\hat{r}_{t} } \right) ^{2} } }}$$

10 $$R_{OOS}^{2} = 1 - \frac{{\mathop \sum \nolimits_{t = 1}^{N} \left( {r_{t + 1} - \hat{r}_{t + 1} } \right)^{2} }}{{\mathop \sum \nolimits_{t = 1}^{N} r_{t + 1}^{2} }}$$

**Table 8:** Results of performance criteria

	Model	MSE	RMSE	MAE	Theil’s U_1_	${R}_{OOS}^{2}$
Bitcoin	M(1)	0.0014	0.0374	0.0255	0.8381	0.0681
	M(2)	0.0014	0.0374	0.0253	0.8286	0.0828
Ethereum	M(1)	0.0027	0.0520	0.0362	0.9434	− 0.0438*
	M(2)	0.0027	0.0520	0.0358	0.9550	− 0.0300*
Cardano	M(1)	0.0037	0.0608	0.0428	0.8131	0.0297
	M(2)	0.0038	0.0616	0.0427	0.9401	0.0043

When evaluating the performance criteria, a lower value of MSE, RMSE, and MAE is preferred in the two models. Theil’s U_1_ value ranges from 0 to 1. If Theil’s U_1_ value is zero, out-of-sample estimations are calculated with the developed model, and the actual values are identical. Therefore, the closer the coefficient is to zero, the more successful the estimate. ${R}_{OOS}^{2}$ values are used to evaluate performance for return predictions.

A comparison of predictions with historical mean returns is commonly used in out-of-sample forecasting applications. When analyzing individual stock returns, this approach is inappropriate, even when it is applied to the aggregate index or long or short portfolio. Forecasting future excess stock returns with historical averages underperforms a naive forecast of zero significantly. As historical mean stock returns are so noisy, they artificially lower the threshold for “good” forecasting performance. R^2^ is benchmarked against the forecast value of zero to avoid this problem (Gu et al 2020).

According to the results presented in Table 8, the MSE and RMSE values of Model 2 for BTC are equal to those of Model 1, while the MAE is lower. Theil’s U_1_ value for Model 2 is lower than that of Model 1, and the ${R}_{OOS}^{2}$ values of Model 1 and 2 are 0.0681 and 0.0828, respectively. According to these results, the prediction model that includes the effect of the day-of-the-week gives better results. The MSE and RMSE values of Model 2 for ETH are equal to those of Model 1, but the MAE value of Model 2 for ETH is lower than that of Model 1. Theil’s U_1_ value for Model 1 is lower than that of Model 2, and the ${R}_{OOS}^{2}$ values of Models 1 and 2 are negative. While the ${R}^{2}$ values from the in-sample are limited to 0 to 1, and the ${R}_{OOS}^{2}$ calculated for the out-of-sample is negative, implying that the model is not compatible with the data. Thus, the model established within the sample was not successful in out-of-sample estimations. As both models established for ETH are meaningless, it means that ETH returns cannot be predicted with ANN. The MSE and RMSE values of Model 2 for ADA are larger than that of Model 1, while the MAE is lower. Theil’s U_1_ value for Model 1 is lower than that of Model 2, and the ${R}_{OOS}^{2}$ values of Model 1 and 2 are 0.0297 and 0.0043, respectively.

The Diebold–Mariano (DM) test compares the forecast accuracy of two forecast methods (Diebold and Mariano 1995; Gu et al. 2020). In this study, the DM test is used to compare Models 1 and 2. Let ${e}_{M(1)}$ and ${e}_{M(2)}$ be the residuals for the two forecasts. ${d}_{i}$ is defined as follows:

11 $$d_{i} = e_{M\left( 1 \right)}^{2} - e_{M\left( 2 \right)}^{2}$$

or

12 $$d_{i} = \left| {e_{M\left( 1 \right)} } \right| - \left| {e_{M\left( 2 \right)} } \right|$$

where ${d}_{i}$ is the loss-differential. Equation 11 is related to *MSE*, and Eq. 12 is related to *MAE*. For $h\ge 1$, the *DM* statistics are as follows:

13 $$DM = \frac{{\overline{d}}}{{\sqrt {\left( {\gamma_{0} + 2\mathop \sum \nolimits_{k = 1}^{h - 1} \gamma_{k} } \right)/n} }}$$

In Eq. 13, $\overline{d }=\frac{1}{n}\sum_{i=1}^{n}{d}_{i}$ and ${\gamma }_{k}$ is the autocovariance at lag *k*. Under the null hypothesis of equal forecast accuracy, *DM* statistics are standard normal distribution. As the *DM* test tends to reject the null hypothesis too often for small samples, the Harvey, Leybourne, and Newbold (HLN) adjustment is made. The HLN test is defined as follows (Harvey et al. 1997):

14 $$HLN = DM\sqrt {\left[ {n + 1 - 2h + h\left( {h - 1} \right)} \right]/n} \sim T\left( {n - 1} \right)$$

The test results for the comparison of Models 1 and 2 are presented in Table 9.

**Table 9:** DM test (HLN adjusted)

	Model class	DM (RMSE)	DM (MAE)
Bitcoin	M(1)_M(2)	6.3466*	26.1493*
Cardano	M(1)_M(2)	− 1.4822	0.1790

*p < 0.001

After the DM test, the null hypothesis was rejected for BTC cryptocurrency but not rejected for ADA cryptocurrency. The forecasts obtained from the two models are significantly different at the 1% level for BTC cryptocurrency, and our findings indicate the existence of the day-of-the-week anomaly.

## Conclusion

Models created with ANNs are used in this study to investigate the effect of the day-of-the-week on crypto prices. After analysis, although suitable network models were established for BTC and ADA, an appropriate network model could not be established for ETH. As the existence of the day-of-the-week anomaly contradicts the weak-form efficient market hypothesis, the results of the analysis confirm that there is no deviation from the weak-form efficient market hypothesis in the ETH market and that it is not possible to predict the future by using the values of the past.

For BTC and ADA, the difference between the predictions of Model 1, which does not include the day-of-the-week effect, and Model 2, which includes the day-of-the-week effect, was investigated with the out-of-sample DM test. According to the DM test results, the difference between the estimates is significant at the 1% level only in BTC. Our results indicate that BTC exhibits the day-of-the-week anomaly.

The existence of the day-of-the-week anomaly indicates that some prices in the BTC markets deviate from the weak-form efficient market hypothesis. Thus, this cryptocurrency contradicts the weak-form efficient market hypothesis. It is possible to forecast the future with past price movements. According to the model results, with Sunday as the base, the highest average return for BTC occurs on Thursday, and the lowest average return occurs on Friday. Investors will be able to determine their strategies according to the returns on these days, allowing them to earn above-normal profits. The analysis reveals that BTC returns were determined to be highest on Thursday. The results of the study by Robiyanto et al. (2019) are consistent with those of this study. Contrary to these results, Durai and Paul (2018) and Evci (2020) determined that the lowest return was on Thursday. Further, contrary to the results of Eyüboğlu (2018) and Nur and Dewangkara (2021), our study found that the lowest BTC returns were on Friday.

In this study, the existence of the day-of-the-week anomaly in cryptocurrency markets was investigated with ANN, which is unlike traditional time series methods, and an evaluation was made from a different perspective. Although evidence of the day-of-the-week anomaly was obtained in this study, the fact that different results are obtained with different methods in the literature indicates that there is still no clear finding on this issue. The crypto money market is not like other markets and maintains its mysterious structure. Any new study that can explain this market will contribute to the literature on this subject.

In addition, as ANN is more successful than classical time series analysis, it can be pointed out that, in this study, more reliable results are obtained about the existence of the day-of-the-week anomaly in cryptocurrencies. Gu et al. (2020) found that neural networks were the best-performing nonlinear method in estimating stock returns, and they emphasized that it was the best estimator in general.

A future study can investigate the effect of the anomaly on specific periods (such as before and after the COVID-19 pandemic or before and after inflationary periods) and different cryptocurrencies. Moreover, a future study may want to use multiple companies in the BTC process to improve the results.

## Data Availability

The data used in the study are secondary published data by coinmarketcap.com, the models or methodology used in the study are not registered. The datasets analysed during the current study are clearly detailed in the text of the paper. Basically: CoinmarketCap (2021) Cryptocurrency Market Capitalizations. https://coinmarketcap.com/tr/. Accessed 06 Sept 2021.

## Abbreviations

ANN: Artificial neural networks
DM: Diebold–Mariano
MSE: Mean squared error
RMSE: Root mean squared error
MAE: Mean absolute error
VAR: Vector autoregression
P2P: Peer-to-peer
BTC: Bitcoin Price Index
ETH: Ethereum Price Index
ADA: Cardano Price Index
BNB: Binance Coin Price Index
USDT: Tether Price Index
XRP: XRP Price Index
SOL: Solana
DOGE: Dogecoin
DOT: Polkadot
USDC: USD coin
COVID-19: Coronavirus
